# Cu_*x*_O/Au chimeric micro–nanoparticles for sensitive SERS monitoring of cigarette smoke *via* synergistic enhancement

**DOI:** 10.1039/d5na01059d

**Published:** 2026-01-05

**Authors:** Yongfeng Tian, Wang Huo, Jing Xie, Shanzhai Shang, Xia Zhang, Donglai Zhu, Gaofeng Dong, Mingquan Yang, Xingjiu Huang, Xianghu Tang

**Affiliations:** a Technology Center of China Tobacco Yunnan Industrial Co., Ltd Kunming 650231 China; b Institute of Solid State Physics, HFIPS, Chinese Academy of Sciences Hefei 230031 China tangxh2011@iim.ac.cn; c Yunnan College of Business Management Kunming 650300 China; d China Tobacco Yunnan Industrial Co., Ltd Kunming 650024 China ymq@ynzy-tobacco.com; e University of Science and Technology of China Hefei 230026 Anhui China

## Abstract

Surface-enhanced Raman spectroscopy (SERS) has been widely used for trace analyte detection, largely depending on the design and fabrication of SERS substrates. Among the various types of substrates, hybrid materials that integrate plasmonic metals with functional semiconductors have attracted increasing attention due to their synergistic properties. This study presents the rational design and synthesis of Cu_*x*_O/Au chimeric micro–nanoparticles as high performance substrates for SERS detection. The hybrids were fabricated *via* a galvanic replacement reaction, where Cu_2_O particles acted as both sacrificial templates and reducing agents for the deposition of Au nanostructures from HAuCl_4_ solutions. By systematically varying the HAuCl_4_ concentration, the morphology, composition, and interfacial properties of the resulting materials were precisely tuned. The optimized substrate, denoted as Cu_*x*_O/Au-10, demonstrated remarkable SERS performance, attributed to the synergistic effect of electromagnetic enhancement from Au nanoparticles and chemical enhancement arising from charge transfer at the metal–semiconductor interface. The SERS activity was rigorously evaluated using nicotine as a probe molecule, showing high detection sensitivity. Furthermore, the practical applicability of the substrate was successfully demonstrated in a dynamic puff-by-puff analysis of tobacco smoke from both conventional cigarettes and heated tobacco products (HNB). SERS detection revealed significantly more complex and intense spectra for traditional cigarette smoke compared to the smoke from HNB cigarettes. This highlights the sensitivity and utility of Cu_*x*_O/Au chimeric micro–nanoparticles for dynamic chemical analysis. This work not only offers a tunable synthesis strategy for high-performance SERS substrates but also paves the way for their application in complex environmental and analytical scenarios.

## Abstract 2

This study developed a series of Cu*_x_*O/Au chimeric micro-nanoparticles exhibiting high effectiveness as SERS substrates for chemical sensing. The optimal Cu_*x*_O/Au-10 substrate was effectively applied in puff-by-puff cigarette smoke analysis.

## Graphical abstract

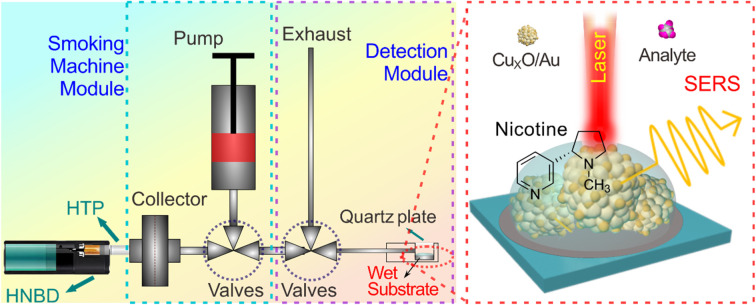

## Introduction

Surface-enhanced Raman spectroscopy (SERS) has emerged as one of the most powerful analytical techniques in molecular spectroscopy, capable of detecting analytes at trace levels by dramatically enhancing the inherently weak Raman signal.^1–3^ This remarkable amplification and sensitivity stems from the tremendous enhancement of the inherently weak Raman scattering signal, which is primarily achieved through two distinct mechanisms: electromagnetic enhancement (EM) and chemical enhancement (CM).^3–5^ The EM mechanism, which often accounts for the majority of the signal enhancement, is a physical phenomenon arising from the localized surface plasmon resonance (LSPR) of noble metal nanostructures, *e.g.*, Au, Ag, Cu. When these nanostructures are irradiated with light of a specific wavelength, collective oscillations of conduction electrons create intensely localized electromagnetic fields, known as “hot spots,” at their surfaces and nanogaps. Molecules residing within these hot spots experience a massive amplification of the incident and scattered light. The CM mechanism, typically contributing a more modest but significant enhancement, involves a charge transfer process between the analyte molecule and the substrate material, often a semiconductor. This process polarizes the analyte molecule, altering its polarizability and thereby increasing its Raman scattering cross-section.

Traditionally, SERS substrates have been dominated by noble metals due to their strong plasmonic properties in the visible and near-infrared regions.^5–7^ However, pure metallic substrates, particularly colloidal nanoparticles, suffer from limitations such as poor reproducibility, aggregation-induced instability, and a lack of chemical specificity. Furthermore, their enhancement is largely confined to the immediate vicinity of the metal surface, which can limit their effectiveness for certain classes of molecules. In recent years, the focus has shifted towards hybrid materials^8–11^ that synergistically combine the strengths of plasmonic metals and functional substrates. The integration of noble metal nanostructures with functional substrates, such as semiconductors, can create a heterojunction interface, which is critical for facilitating efficient charge transfer, thereby potentially unlocking a new level of SERS sensitivity and selectivity.^12–15^

Among various metal–oxide nanomaterials, Cu_2_O is a particularly attractive candidate for constructing hybrid SERS substrates. As a p-type semiconductor with a direct bandgap, the utility of Cu_2_O in SERS, however, is limited when used alone because its CM effect is relatively weak compared to the EM of noble metals.^16–18^ This has led to the exploration of hybrid structures combining Cu_2_O with plasmonic metals like Au nanostructures. The concept is to create a substrate where the intense EM fields generated by Au nanoparticles are complemented by the efficient charge-transfer pathways provided by the semiconductor, potentially leading to a synergistic enhancement that surpasses the capabilities of either component alone.^19–21^ A highly effective strategy for constructing such hybrid structures is the galvanic replacement reaction. Cu_2_O can serve as a sacrificial template and a reducing agent in galvanic replacement reactions with metal precursors such as HAuCl_4_. The standard reduction potential of the AuCl_4_^−^/Au pair (≈0.99 V *vs.* SHE) is significantly higher than that of the Cu^2+^/Cu (≈0.34 V *vs.* SHE) and Cu^2+^/Cu^+^ pairs, making the galvanic replacement reactions thermodynamically spontaneous. This reaction allows for the *in situ* deposition of Au nanostructures onto the Cu_2_O surface, simultaneously transforming part of the Cu_2_O into a composite copper oxide (Cu_*x*_O, where *x* = 1, 2), thereby forming a unique chimeric micro–nano-architecture, which is not a simple core–shell structure but rather an integrated, heterogeneous material where the metallic and semiconducting phases coexist intimately. This intrinsic heterogeneity is key to generating a high density of metal–semiconductor interfaces, which has potential for high performance in SERS detection.

Based on the above analysis, this study is dedicated to the rational design, systematic synthesis, and thorough evaluation of such Cu_*x*_O/Au chimeric micro–nanoparticles as high-performance SERS substrates. The central hypothesis is that by precisely controlling the degree of galvanic replacement through the concentration of HAuCl_4_, one can fine-tune the morphology, composition, and interfacial properties of the resulting hybrid material. This tunability is anticipated to directly govern the SERS activity by optimizing the synergy between the plasmonic Au nanoparticles and the semiconducting Cu_*x*_O matrix. Subsequently, to demonstrate the practical utility and superior sensitivity of the optimized chimeric micro–nanoparticles, they were deployed in a sophisticated, real-world application: the puff-by-puff analysis of tobacco smoke from traditional cigarettes and heated tobacco products (HNB). As a type of new tobacco product, HNB heats the tobacco matrix through precise temperature control (usually 250–350 °C) to release nicotine-containing aerosols instead of burning smoke. Studies have shown that the release of harmful components (such as TSNAs, aldehydes, *etc.*) in HNB aerosols is more than 80% lower than that of traditional cigarettes.^22–24^ This significant harm reduction has made it quickly popular in markets such as Japan, replacing more than 30% of traditional cigarettes.^25^ However, while there is a consensus that smoking is detrimental to health,^26–28^ many questions remain regarding new tobacco products like HNB, even though they are marketed as harm reduction alternatives. The harm reduction effect of HNB cigarette products is highly dependent on the stability and controllability of ingredient release. At present, HNB ingredient detection mainly relies on chromatography-mass spectrometry (GC-MS/LC-MS), which has high accuracy (the detection limit reaches the ppb level),^29,30^ but has significant defects: complex sample pretreatment, *e.g.*, extraction, enrichment and other steps are required, which destroys *in situ* information. In contrast, optical and spectral analysis, such as SERS technology, has shown significant advantages in the field of material detection due to its unique molecular fingerprint recognition ability.^31–33^ Thus, the study powerfully demonstrates the practical utility of the optimized Cu_*x*_O/Au substrate by applying it to a challenging real-world analysis: the puff-by-puff SERS detection of tobacco smoke from traditional and HNB cigarettes. The dynamic detection setup, utilizing a PTFE membrane loaded with the substrate, successfully captures the temporal evolution of smoke constituents. This study involved a dynamic analysis methodology, which tracks chemical changes throughout the consumption process, and provided a rigorous test for the substrate's performance. What's more, the application in analyzing complex aerosols like tobacco smoke validates the great promise of these chimeric micro–nanoparticles. They serve not only as highly efficient and tunable SERS substrates but also as a model system for understanding the synergistic enhancement of metal–semiconductor interfaces. This research opens up new avenues for the development of advanced sensing platforms for environmental monitoring.

## Experimental

### Materials

Cu(CH_3_COO)_2_·H_2_O was purchased from Aladdin, Shanghai, Co. N_2_H_4_·H_2_O, HAuCl_4_, Crystal Violet (CV) and Methyl Blue (MB) were purchased from Shanghai Chemicals Co. Cigarettes were provided by China Tobacco Yunnan Industrial Co., Ltd. All experimental glassware was washed with aqua regia before use. All reagents were of analytical grade and used without further purification. Milli-Q deionized water (18.2 MΩ cm) was used for all preparations.

### Synthesis of Cu_2_O

The synthesis was carried out according to the literature^34^ with a slight modification. A typical experiment to synthesize Cu_2_O was as follows: the preparation method is as follows: 0.2 g Cu(CH_3_COO)_2_·H_2_O is added to a beaker containing 40 mL H_2_O, and slowly stirred at room temperature for 5 min to disperse it evenly, and the solution finally turns blue. Then, 200 µL N_2_H_4_·H_2_O is immediately and quickly injected, and stirring is continued for 30 min. The solution turns from blue to yellow, and the stirred stock solution is poured into a 50 mL polypropylene centrifuge tube at a speed of 8000 rpm for 5 min. The supernatant is poured out of the centrifuge tube, and the residue is redispersed in solvent to the original volume, centrifuged, dispersed again, and finally placed in a 60 °C vacuum drying oven for 12 h.

### Preparation of Cu_*x*_O/Au chimeric micro–nanoparticles

The chimera of Au and Cu_*x*_O in different proportions was achieved by adding HAuCl_4_ of different concentrations. The specific preparation method is as follows: 5 mg of Cu_2_O was dispersed in 5 mL of deionized water (1 mg mL^−1^), ultrasonicated, and the above solution was poured into a beaker containing 20 mL of deionized water, ultrasonicated and stirred to obtain solution A. 1.25 mL of HAuCl_4_ of different concentrations (0.25, 2.5, 5, 10, 15 mM) was added to 20 mL of deionized water, and solution B was obtained by ultrasonication. Solution B was poured into solution A and stirred continuously for 20 min. The final stock solution was poured into a 50 mL polypropylene centrifuge tube, centrifuged at 8000 rpm for 5 min, the supernatant was discarded, and the solution was redispersed to the original volume, and finally placed in a 60 °C vacuum drying oven for 12 h.

### Evaluation of SERS performance of Cu_*x*_O/Au micro–nanoparticles

The SERS effect of Cu_*x*_O/Au chimeric micro–nanoparticles was evaluated as follows: take 1 mL of Cu_*x*_O/Au dispersion, concentrate it to 100 µL by centrifugation, take 10 µL of the concentrate and place it into 1.5 mL centrifuge tubes to divide it into several tubes. Then add probe molecule solutions to 1 mL volume, shake for 30 s and let it stand for 5 min. Then centrifuge at 8000 rpm for 10 min, discard the supernatant, keep 100 µL of the concentrate, and finally take 10 µL of the dispersion drop on the polytetrafluoroethylene (PTFE) membrane for a Raman test.

## Results and discussion

**Fig. 1:**
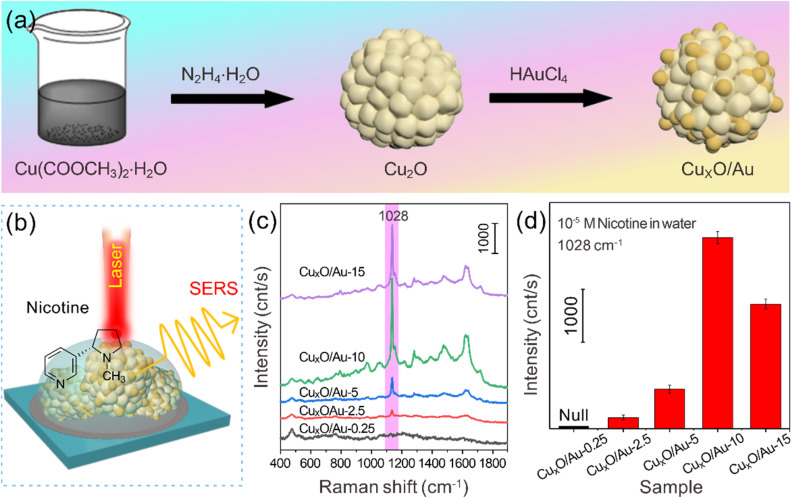
(a) Schematic diagram of the design principle and preparation process flow of Cu_*x*_O/Au chimeric micro–nanoparticles. (b) Schematic diagram of SERS detection with Cu_*x*_O/Au as substrates. (c) SERS spectra of nicotine on Cu_*x*_O/Au-0.25, Cu_*x*_O/Au-2.5, Cu_*x*_O/Au-5, Cu_*x*_O/Au-10 and Cu_*x*_O/Au-15 substrates, respectively. (d) Comparison of SERS signal intensities at 1028 cm^−1^ for nicotine molecules detected based on different substrates.

Fig. 1 presents a schematic illustration of the design principle and preparation process flow for the Cu_*x*_O/Au chimeric micro–nanoparticles. The process was initiated with the synthesis of Cu_2_O particles through a reduction reaction, where Cu(CH_3_COO)_2_·H_2_O was reduced by N_2_H_4_·H_2_O in an aqueous solution. This resulted in a visible color change from blue to yellow, indicating the successful formation of Cu_2_O particles. Subsequently, these particles served as both templates and reducing agents for the deposition of Au nanoparticles through a galvanic replacement reaction when exposed to HAuCl_4_ solutions of varying concentrations. This step was critical for forming the chimeric structures, as the Cu_2_O cores partially reduce Au^3+^ to Au^0^ (the reaction principle is shown in formula (1)), leading to part of the monovalent copper being oxidized to its divalent form to form a Cu_*x*_O composite in which Cu_2_O and CuO coexist, which resulted in the formation of Au nanostructures anchored onto or embedded within the oxide matrix, thereby forming Cu_*x*_O/Au chimeric micro–nanoparticles. The reaction equation for the process can be expressed as follows:

1 3Cu_2_O + 6H^+^ + 2AuCl_4_^−^ → 2Au + 6Cu^2+^ + 8Cl^−^ + 3H_2_O

This galvanic replacement-based synthetic route allowed for precise control over the composition and morphology of the resulting hybrid material by simply adjusting the concentration of HAuCl_4_. The final products were denoted as Cu_*x*_O/Au-0.25, Cu_*x*_O/Au-2.5, Cu_*x*_O/Au-5, Cu_*x*_O/Au-10, and Cu_*x*_O/Au-15, respectively, corresponding to the different molar concentrations of HAuCl_4_ used (0.25 mM to 15 mM).

In order to explore the relationship between the chimeric micro–nanoparticle regulation process and SERS performance, the nicotine standard solution with a concentration of 10^−5^ M was selected as the probe molecule to evaluate the SERS activity of Cu_*x*_O/Au chimeras for constructing substrates. Fig. 1b schematically demonstrates the SERS detection employing Cu_*x*_O/Au chimeric particles as substrates. The significantly enhanced Raman signals can be attributed to the combined effects of electromagnetic enhancement (primarily from Au nanoparticles) and chemical enhancement (related to charge transfer between the analyte and the semiconductor oxide).^20,21^ The Au nanostructures generate strong LSPR, creating highly enhanced electromagnetic fields around their surfaces. Meanwhile, the Cu_*x*_O substrate may facilitate analyte adsorption *via* electrostatic interaction or formation of coordination bonds, further amplifying the SERS signal through a chemical enhancement mechanism. This dual enhancement makes the hybrid material particularly effective for detecting low-concentration analytes.

Fig. 1c displays representative SERS spectra of nicotine adsorbed on different substrates. The presence of distinct Raman peaks of nicotine confirmed the SERS activity of the Cu_*x*_O/Au substrates. The enhancement in Raman signals can be attributed to the synergistic effect between the plasmonic Au nanoparticles and the semiconducting Cu_*x*_O substrates. The spectra show characteristic Raman peaks of nicotine, among which the signal at 1028 cm^−1^ is particularly prominent and is assigned to the stretching vibrations of the pyridine ring of nicotine.^31–33,35^ It is evident that the intensity of the Raman signals varies significantly with the amount of Au incorporated. No SERS signal for nicotine was observed when using pure Cu_2_O substrates (lacking Au nanoparticles) or those with an extremely low Au content, such as Cu_*x*_O/Au-0.25. This result indicates that Au nanoparticles play a crucial role in the SERS detection of nicotine, likely due to insufficient density of Au nanoparticles and hence limited plasmonic enhancement. As the concentration of Au nanoparticles increases, the SERS intensity strengthens, reaching an optimum range. Fig. 1d presents a comparative analysis of the SERS signal intensities recorded at the characteristic Raman shift of 1028 cm^−1^ for nicotine, across the different chimeric substrates by Cu_*x*_O/Au-0.25, Cu_*x*_O/Au-2.5, Cu_*x*_O/Au-5, Cu_*x*_O/Au-10, and Cu_*x*_O/Au-15, respectively. The results clearly demonstrated that the SERS performance was highly dependent on the amount of Au incorporated into the structure. Among the series, Cu_*x*_O/Au-10 likely exhibited the highest intensity, indicating an optimal balance between Au loading and Cu_*x*_O surface coverage, and the density and spacing of Au nanoparticles may reach an ideal state, generating the strongest electromagnetic field enhancement; meanwhile, the semiconductor properties of Cu_*x*_O may produce a synergistic coupling effect with Au, further improving the charge transfer efficiency. With Cu_*x*_O/Au-15 as the substrate, the signal intensity is reduced compared to that with Cu_*x*_O/Au-10 as the substrate, indicating that excessive Au causes nanoparticles to agglomerate or cover the Cu_*x*_O active sites, while the semiconductor properties of Cu_*x*_O and Au produce a synergistic coupling effect, resulting in a decrease in the performance, which reduces the density of “hot spots” or hinders charge transfer. The above results indicate that when the chimeric micro–nanoparticles formed by Au loading and Cu_*x*_O in a suitable ratio are used as SERS substrates, Cu_*x*_O as a carrier not only stabilizes the nano-Au, but also activates its own chemical enhancement contribution (such as metal–semiconductor interface charge transfer), so that the Cu_*x*_O/Au-10 sample exhibits excellent SERS activity due to its optimized nanostructure and synergistic enhancement mechanism. Broadly speaking, Fig. 1 comprehensively illustrates the rational design, fabrication, and functional evaluation of Cu_*x*_O/Au chimeric micro–nanoparticles. The results confirm that these chimeric micro–nanoparticles serve as highly efficient SERS substrates, with performance tunable through synthetic parameters, offering great promise for advanced chemical sensing applications. For instance, the successful detection of nicotine suggests potential use in environmental monitoring or tobacco-related research.

**Fig. 2:**
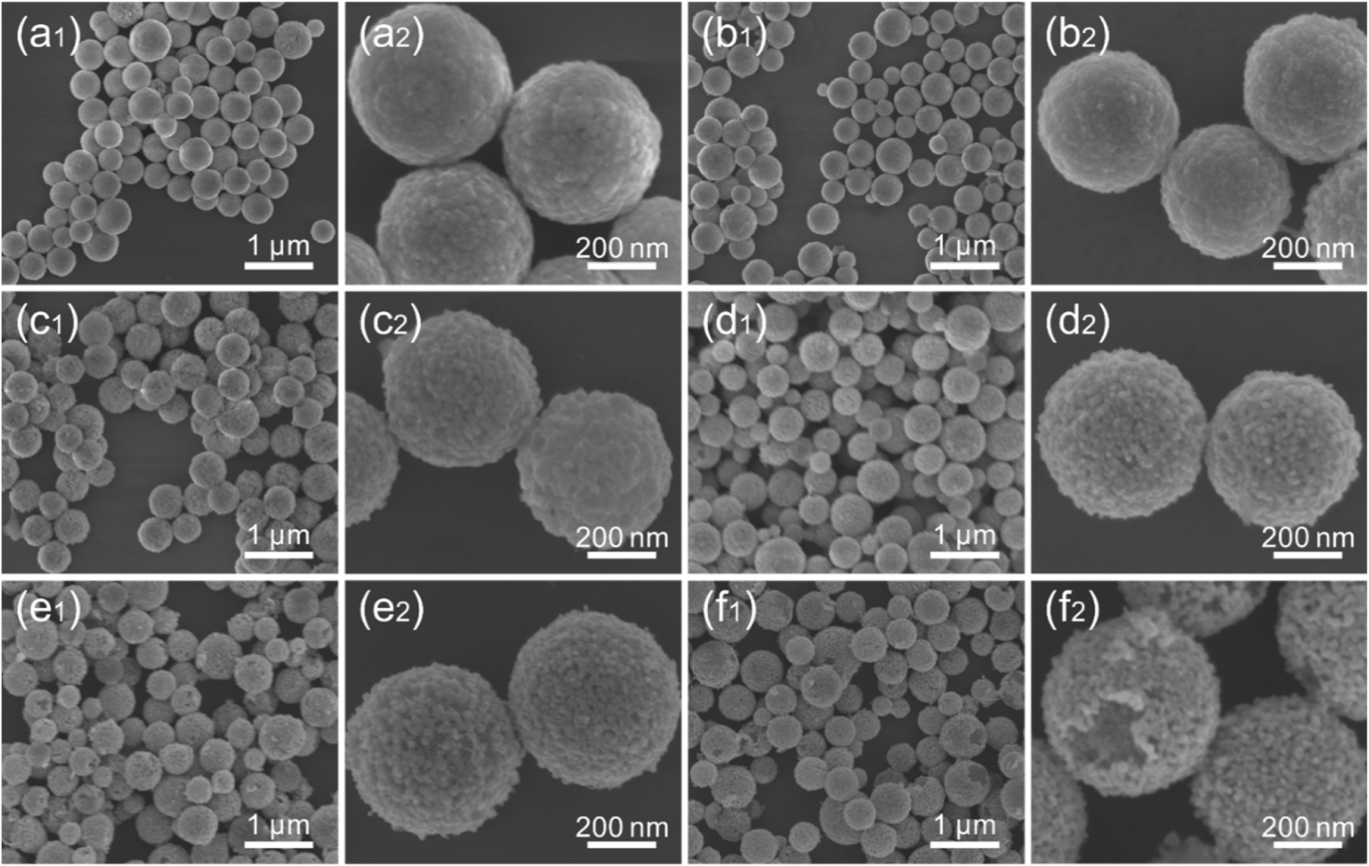
SEM images of (a) Cu_2_O, (b) Cu_*x*_O/Au-0.25, (c) Cu_*x*_O/Au-2.5, (d) Cu_*x*_O/Au-5, (e) Cu_*x*_O/Au-10, and (f) Cu_*x*_O/Au-15, respectively.

For the Cu_*x*_O/Au series of substrates, the variations in their structure and properties, particularly the enhancement of SERS activity, arise from the fine-tuning of their internal morphology and architecture due to the integration of Cu_*x*_O and Au. The corresponding morphological and structural evolution is key to understanding the sample properties. Fig. 2 lists a series of SEM images illustrating the morphological evolution of Cu_2_O and Cu_*x*_O/Au chimeric micro–nanoparticles, including Cu_*x*_O/Au-0.25, Cu_*x*_O/Au-2.5, Cu_*x*_O/Au-5, Cu_*x*_O/Au-10, and Cu_*x*_O/Au-15, respectively. These images provide critical insights into the structural modifications and composite formation processes resulting from the incorporation of Au onto the Cu_*x*_O framework. As shown in Fig. 2a, the pristine Cu_2_O particles exhibit a relatively uniform polyhedral structure with clear edges and smooth surfaces, typical of chemically synthesized cuprous oxide crystals. The morphology suggests a well-defined crystalline nature, which is consistent with the reduction process involving N_2_H_4_·H_2_O. With the introduction of a low concentration of HAuCl_4_ (0.25 mM), denoted as Cu_*x*_O/Au-0.25, as can be seen in Fig. 2b, the underlying Cu_*x*_O structure remains largely intact, though some surface roughening is observable. As the HAuCl_4_ concentration increases to 2.5 mM, significant changes in morphology become apparent, as can be seen in Fig. 2c. Small particulate deposits, likely metallic Au, begin to appear on the surface of the Cu_*x*_O particles. This indicates galvanic replacement leading to the nucleation of Au nanoparticles on the oxide surface. As the HAuCl_4_ concentration increases to 5 mM (Cu_*x*_O/Au-5), as shown in Fig. 2d, it can be seen that there is more pronounced deposition and possible alloying or interfacial diffusion between Au and Cu_*x*_O. The particles show increased aggregation and surface heterogeneity. The Cu_*x*_O surfaces become more decorated with Au domains, suggesting progressive replacement and deposition. The core–shell type structure begins to emerge, though the original morphology of Cu_*x*_O is still recognizable. At higher concentrations of HAuCl_4_ (10 mM), which resulted in the formation of Cu_*x*_O/Au-10 (Fig. 2e), the transformation becomes extensive. The sharp edges of the initial Cu_*x*_O are now less distinct, implying surface reconstruction or partial dissolution followed by re-deposition. This suggests extensive coverage and growth of Au, likely through both displacement and reduction processes. The chimeric structure is prominently visible, with the particles adopting a more irregular and aggregated profile due to the increased Au loading. The formation of such chimeric structures may enhance SERS activity due to synergistic effects between Au and Cu_*x*_O. When the concentration of HAuCl_4_ was increased to 15 mM, the original Cu_*x*_O morphology became extensively obscured by dense, cauliflower-like Au nanostructures, as shown in Fig. 2f. Accompanying the substantial consumption of Cu_*x*_O, portions of the Cu_*x*_O/Au-15 composite developed into non-compact cavities or open porous frameworks. This degradation of the structural integrity in certain Cu_*x*_O/Au micro–nanoparticles consequently impaired the performance of the material when employed as a SERS substrate. Overall, the above morphological evolution is expected to directly influence the SERS activity of the materials, providing a foundation for understanding the functional application studies of the Cu_*x*_O/Au series substrates corresponding to Fig. 1. Furthermore, Fig. 2 and S1 (more details can be seen in Fig. S1 and S2, SI) show the morphological and compositional evolution of the Cu_*x*_O/Au series substrates, highlighting the importance of optimizing synthesis conditions to achieve a balance between Au integration and structural preservation for maximum functional efficacy.

**Fig. 3:**
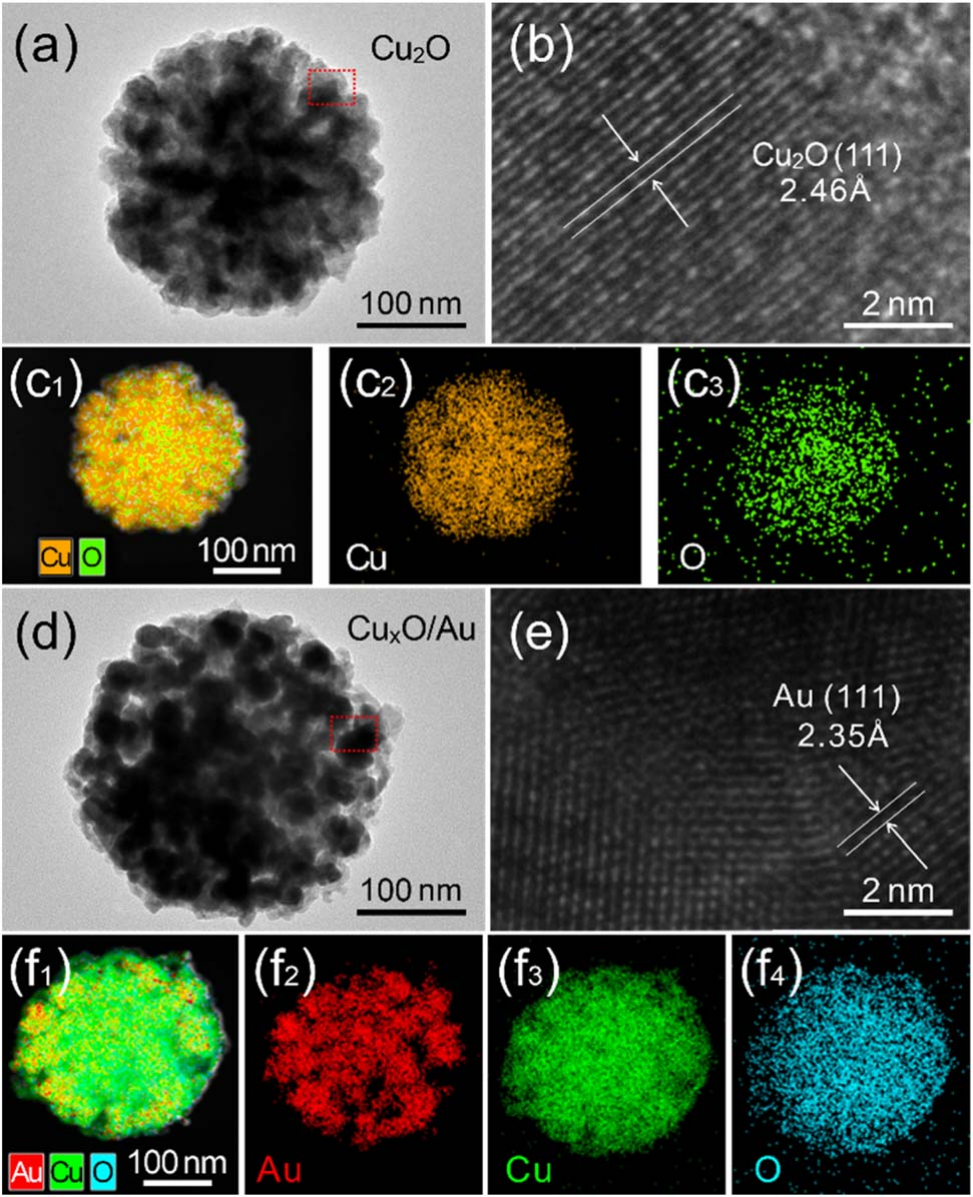
(a–c) TEM and HRTEM images, and EDS mapping of a single Cu_2_O micro–nanoparticle. (d–f) TEM and HRTEM images, and EDS mapping of a single Cu_*x*_O/Au-10 micro–nanoparticle.

Furthermore, the contrasting structural characteristics between pure Cu_2_O and Cu_*x*_O/Au-10 micro–nanoparticles were revealed through detailed TEM and HRTEM analysis. Fig. 3a presents a representative TEM image of a single Cu_2_O particle, revealing a solid, polyhedral structure, which is typical of crystalline cuprous oxide synthesized *via* chemical reduction methods. Further insights into the crystallinity of the as-synthesized Cu_2_O are provided by the HRTEM image in Fig. 3b. The clear and distinct lattice fringes are a hallmark of a highly crystalline material. The measured interplanar spacing can be accurately correlated with the known *d*-spacings of cubic Cu_2_O, *e.g.*, the (110) crystal plane, confirming the phase purity of the initial product. The corresponding Energy-Dispersive X-ray Spectroscopy (EDS) mapping in Fig. 3c provides elemental verification, showing a uniform distribution of Cu and O elements throughout the particle, with no detectable impurities. This homogeneous signal confirms the composition of the particle as pure Cu_2_O. Meanwhile, the architectural evolution upon the introduction of Au was strikingly evident in the chimeric Cu_*x*_O/Au-10 micro–nanoparticles, as depicted in Fig. 3d–f. The galvanic replacement reaction, driven by the standard reduction potential difference between the AuCl_4_^−^/Au and Cu^2+^/Cu pairs, leads to a significant morphological transformation. The TEM image in Fig. 3d shows a stark contrast to the pristine Cu_2_O. The originally smooth surface of the Cu_2_O particle becomes decorated with smaller, darker contrasted nanoparticles. These darker features are indicative of the formation of metallic Au nanostructures around Cu_*x*_O, creating a heterogeneous, chimeric architecture. The HRTEM image of the Cu_*x*_O/Au-10 particle (Fig. 3e) offers compelling evidence for the coexistence of multiple distinct crystalline phases. The image reveals multiple sets of lattice fringes with different interplanar spacings. One set corresponds to the expectations for metallic Au, *e.g.*, the (111) plane of face-centered cubic Au. The interface between these crystalline domains confirms the formation of a well-defined heterojunction, which is crucial for facilitating charge transfer between the semiconductor Cu_*x*_O and the metal Au. This intimate contact is a key factor in enhancing the functional properties of the material. The most definitive proof of the successful chimeric formation comes from the EDS elemental mapping presented in Fig. 3f. The maps for Cu and O show a distribution that outlines the larger micro–nanoparticle core. Crucially, the Au map reveals a highly specific signal that overlaps precisely with the darker nanoparticle domains observed in the TEM image. This spatially resolved elemental analysis confirms that the chimeric structure consists of a Cu_*x*_O adorned with Au nanostructures.

**Fig. 4:**
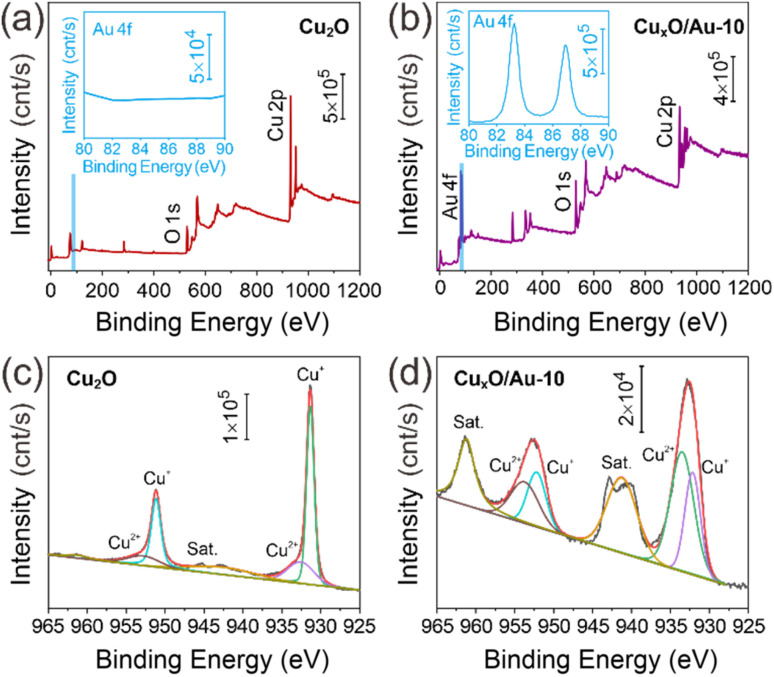
XPS analysis of the synthesized materials. (a) Full survey spectrum of pure Cu_2_O. (b) Full survey spectrum of the Cu_*x*_O/Au-10 micro–nanoparticles. The insets in (a) and (b) provide enlarged views of the 80–90 eV region, highlighting the Au 4f signals. (c) High-resolution Cu 2p spectrum of the pure Cu_2_O. (d) High-resolution Cu 2p spectrum of the Cu_*x*_O/Au-10 micro–nanoparticles.

To further explore the surface structure and composition of the material, XPS spectra of Cu_2_O and Cu_*x*_O/Au-10 chimeric micro–nanoparticles were analyzed, and the results are shown in Fig. 4, which presents a comprehensive set of XPS data, including the full survey scans and high-resolution regional spectra, which provide critical insights into the chemical states and interfacial interactions within the materials. The spectrum for pure Cu_2_O (Fig. 4a) shows prominent peaks corresponding to Cu and O, as expected. In stark contrast, the survey spectrum of the Cu_*x*_O/Au-10 composite (Fig. 4b) clearly reveals the presence of additional peaks. The most significant of these are the signals for Au, which are conspicuously absent in the pure Cu_2_O sample. This direct elemental evidence confirms the successful integration of Au into the composite structure. The insets in these figures, which magnify the binding energy region between 80 and 90 eV, are particularly revealing. This region is where the Au 4f peaks are located. The inset in Fig. 4a shows a flat baseline, confirming no detectable Au in the pure sample. Conversely, the inset in Fig. 4b displays distinct doublet peaks, characteristic of the spin–orbit split Au 4f_7/2_ and Au 4f_5/2_ states,^36–38^ providing undeniable proof of the presence of metallic Au in the Cu_*x*_O/Au-10 chimeric micro–nanoparticles. To gain a deeper understanding of the chemical environment and potential electronic interactions between the components, high-resolution scans of the Cu 2p region were performed and are displayed in Fig. 4c and d. The Cu 2p spectrum of the pure Cu_2_O sample (Fig. 4c) exhibits two main peaks around 932.1 eV and 952.0 eV, which are assigned to the Cu 2p_3/2_ and Cu 2p_1/2_ of monovalent copper,^38–40^ respectively. This is a classic signature of Cu_2_O. Furthermore, the absence of significant satellite features in the higher binding energy region (940–945 eV) is a key indicator. These “shake-up” satellites are a hallmark of Cu^2+^ species (as in CuO). Their absence strongly suggests that the synthesized material is predominantly Cu_2_O, with minimal surface oxidation to CuO, which is a common challenge in the handling of cuprous oxide. The corresponding Cu 2p spectrum for the Cu_*x*_O/Au-10 (Fig. 4d) reveals subtle but crucial shifts. While the peak shapes and the absence of strong satellites confirm that Cu^+^ remains the dominant copper species, a careful comparison shows a slight shift in the binding energy of the Cu 2p peaks. Typically, a small but measurable positive shift (towards higher binding energy) is observed. This shift is highly significant as it suggests an electronic interaction at the interface between the Cu_*x*_O and Au phases. When metallic Au, which has a higher work function, comes into contact with a p-type semiconductor like Cu_2_O, electron transfer occurs from Cu_2_O to Au to equilibrate the Fermi levels. This loss of electron density from the copper atoms results in a slight increase in the binding energy required to eject a core electron during XPS analysis. This phenomenon provides strong evidence that the composite is not merely a physical mixture but features a chemically interactive interface, which is crucial for enhancing SERS activity.

**Fig. 5:**
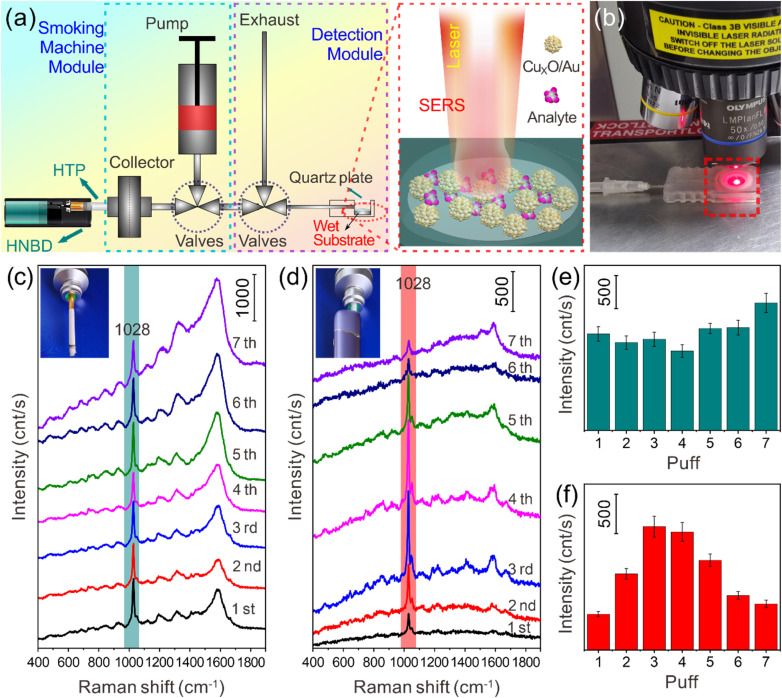
SERS-based analysis of tobacco smoke from traditional and HNB cigarettes. (a) Schematic illustration of the experimental setup using a PTFE membrane loaded with Cu_*x*_O/Au-10 chimeric micro–nanoparticles for SERS detection. (b) Representative photograph of the actual SERS detection setup used for analyzing captured tobacco smoke. (c) SERS spectra obtained from puff-by-puff analysis of traditional cigarette smoke. (d) SERS spectra obtained from puff-by-puff analysis of HNB cigarette smoke. (e) Comparison of the SERS intensities at the 1028 cm^−1^ peak corresponding to the spectra in (c). (f) Comparison of the SERS intensities at the 1028 cm^−1^ peak corresponding to the spectra in (d).

The above studies show that among the prepared series of Cu_*x*_O/Au chimeric micro–nanoparticles, Cu_*x*_O/Au-10 exhibits excellent SERS activity (more details can be seen in Fig. S3 and S4, SI) due to its optimized nanostructure and synergistic enhancement mechanism, so it is used in subsequent application research. Studies have shown that nicotine-containing products, whether combustible cigarettes or smokeless products, pose a growing threat to cardiovascular health.^41–43^ While smoking continues to cause millions of deaths annually, the rapid uptake of e-cigarettes, HNB cigarettes, and synthetic nicotine pouches such as snus, risks reversing decades of progress in tobacco control.^41^ Therefore, the development of a rapid detection method for nicotine based on SERS technology holds practical value for the regulatory monitoring of nicotine release. To better perform SERS monitoring of cigarette smoke, a series of devices or modules were designed for the research (Fig. S5, SI). The efficacy of the synthesized Cu_*x*_O/Au chimeric micro–nanoparticles as substrates for SERS is compellingly demonstrated in Fig. 5, which details a comparative analysis of smoke constituents from traditional cigarettes and HNB cigarettes. The experimental methodology is visually summarized in Fig. 5a and b. Fig. 5a provides a schematic diagram of the detection platform, where the Cu_*x*_O/Au-10 micro–nanoparticles are deposited onto a PTFE membrane. This membrane acts as a robust and inert support for capturing tobacco smoke. The integration of the plasmonic Au nanoparticles with the semiconductor Cu_*x*_O is crucial here, as it creates “hot spots” that significantly enhance the Raman signal of molecules of tobacco smoke adsorbed on the surface. The smoke is introduced into the microflow channel through a pipe. Typically, a PTFE membrane loaded with Cu_*x*_O/Au-10 chimeric micro–nanoparticles is pre-installed at the end of the channel (the membrane is cut to a size of 0.6 mm × 0.6 mm and encapsulated in the channel pool. The top of the pool has a removable quartz plate cover and a micro vent hole). When conducting the experiment, first take 100 µL of water to wet the membrane, then place the channel and detection pool module on the Raman spectrometer workbench, adjust the optical path system of the Raman spectrometer and the focusing state of the workbench objective lens, then start simulating smoking, introduce smoke into the channel, and then perform SERS detection, with an excitation light wavelength of 633 nm. Fig. 5b shows a representative photograph of the actual setup, confirming the practical feasibility of the schematic. The core findings of the study are presented in Fig. 5c and d, which display the SERS spectra acquired from a puff-by-puff analysis. This analytical approach is particularly sophisticated, as it allows for the tracking of chemical changes throughout the consumption of a single cigarette, rather than providing a single, averaged measurement. It is noted that a typical cigarette is consumed in 6 to 12 puffs, making this high-resolution analysis highly relevant. Fig. 5c showcases the SERS spectra for each puff from a traditional cigarette. The spectra are characterized by multiple distinct peaks, indicating a complex mixture of chemical compounds present in the smoke. In stark contrast, Fig. 5d presents the SERS spectra for the HNB cigarette. A visual comparison immediately reveals a significant reduction in the number and intensity of Raman peaks across all puffs. This stark difference suggests a substantially lower concentration and diversity of chemical species in the aerosol generated by the HNB product compared to the smoke from traditional combustion. To provide a quantitative dimension to the spectral data, panels (e) and (f) of Fig. 5 focus on the intensity of a specific Raman peak at 1028 cm^−1^. This peak corresponds to a characteristic vibrational mode of the nicotine captured from the smoke. The choice of a single, well-defined peak for quantification is a useful practice for simplifying complex spectral data and enabling direct comparison. Fig. 5e plots the intensity of this peak for each puff of a traditional cigarette. The trend may show variations, potentially indicating fluctuations in the concentration of the target molecule during smoking. Conversely, Fig. 5f presents the same quantitative analysis for the HNB cigarette. The signal intensities in Fig. 5f are expected to be consistently and significantly lower than those observed in Fig. 5e, quantitatively confirming the visual observation from the spectra. Overall, the data presented in Fig. 5 indicate that the successful application of the Cu_*x*_O/Au chimeric micro–nanoparticles validates their superior performance as sensitive and reliable SERS substrates. The puff-by-puff analysis methodology demonstrates a high temporal resolution for dynamic chemical processes. Most importantly, the comparative results between traditional and HNB cigarettes provide tangible evidence of fundamental differences in their emissions. The significantly weaker SERS signals from the HNB product suggest a reduction in the concentration of certain harmful or characteristic constituents, which could be attributed to the absence of pyrolysis and combustion in HNB technology. This finding has substantial implications for understanding the relative chemical exposures associated with different tobacco products. Further research could focus on identifying the specific molecules corresponding to the observed Raman peaks to gain deeper insights into the toxicological profiles of these aerosols.

## Conclusions

This study successfully developed a series of Cu_*x*_O/Au chimeric micro–nanoparticles *via* a galvanic replacement reaction and demonstrated their high effectiveness as SERS substrates for chemical sensing. The synthesis involved using Cu_2_O particles as both templates and reducing agents to deposit Au nanostructures from HAuCl_4_ solutions of varying concentrations. Comprehensive characterization using SEM, TEM, HRTEM, and XPS confirmed the successful formation of chimeric structures. The SERS performance was highly dependent on the Au loading. An optimal balance was achieved with the Cu_*x*_O/Au-10 substrate, which exhibited the strongest enhancement due to a synergistic effect between the plasmonic Au nanoparticles (providing electromagnetic enhancement) and the semiconductor Cu_*x*_O (providing chemical enhancement *via* charge transfer). Moreover, the practical application of the optimal Cu_*x*_O/Au-10 substrate was convincingly demonstrated in a puff-by-puff analysis of cigarette smoke. SERS detection revealed significantly more complex and intense spectra for traditional cigarette smoke compared to the smoke from HNB cigarettes. This highlights the sensitivity and utility of Cu_*x*_O/Au chimeric micro–nanoparticles for dynamic chemical analysis. In conclusion, this work presents a tunable synthesis strategy for efficient SERS substrates and validates their potential for advanced sensing applications.

## Conflicts of interest

There are no conflicts of interest to declare.

## Supplementary Material

NA-008-D5NA01059D-s001

## Data Availability

The data supporting this article have been included as part of the supplementary information (SI). Supplementary information: ESI_1 and Fig. S1–S5. See DOI: https://doi.org/10.1039/d5na01059d.
