# Supplementary material for: CuxO/Au chimeric micro–nanoparticles for sensitive SERS monitoring of cigarette smoke via synergistic enhancement
Source: Nanoscale Adv. 2026 Jan 5;8(4):1400–8. doi: 10.1039/d5na01059d (PMC12834215; doi:10.1039/d5na01059d)
Supplement: NA-008-D5NA01059D-s001 [file NA-008-D5NA01059D-s001.pdf]

Supplementary Information (SI) for Nanoscale Advances.
This journal is © The Royal Society of Chemistry 2026

Electronic Supplementary Information (ESI)

Cu_xO/Au Chimeric Micro-Nanoparticles for Sensitive SERS Monitoring of Cigarette Smoke via Synergistic Enhancement†

Yongfeng Tian[a], Wang Huo[b], Jing Xie[c], Shanzhai Shang[a], Xia Zhang[a], Donglai Zhu[a], Gaofeng Dong[a], Mingquan Yang[d*], Xingjiu Huang[b,e], Xianghu Tang[b,e*]

a. Technology Center of China Tobacco Yunnan Industrial Co., Ltd., Kunming 650231, China.

b. Institute of Solid State Physics, HFIPS, Chinese Academy of Sciences, Hefei 230031, China.

c. Yunnan College of Business Management,Kunming 650300, China

d. China Tobacco Yunnan Industrial Co., Ltd, Kunming 650024, China.

e. University of Science and Technology of China, Hefei 230026, Anhui, China.

*Corresponding authors.

E-mail: tangxh2011@iim.ac.cn; ymq@ynzy-tobacco.com.

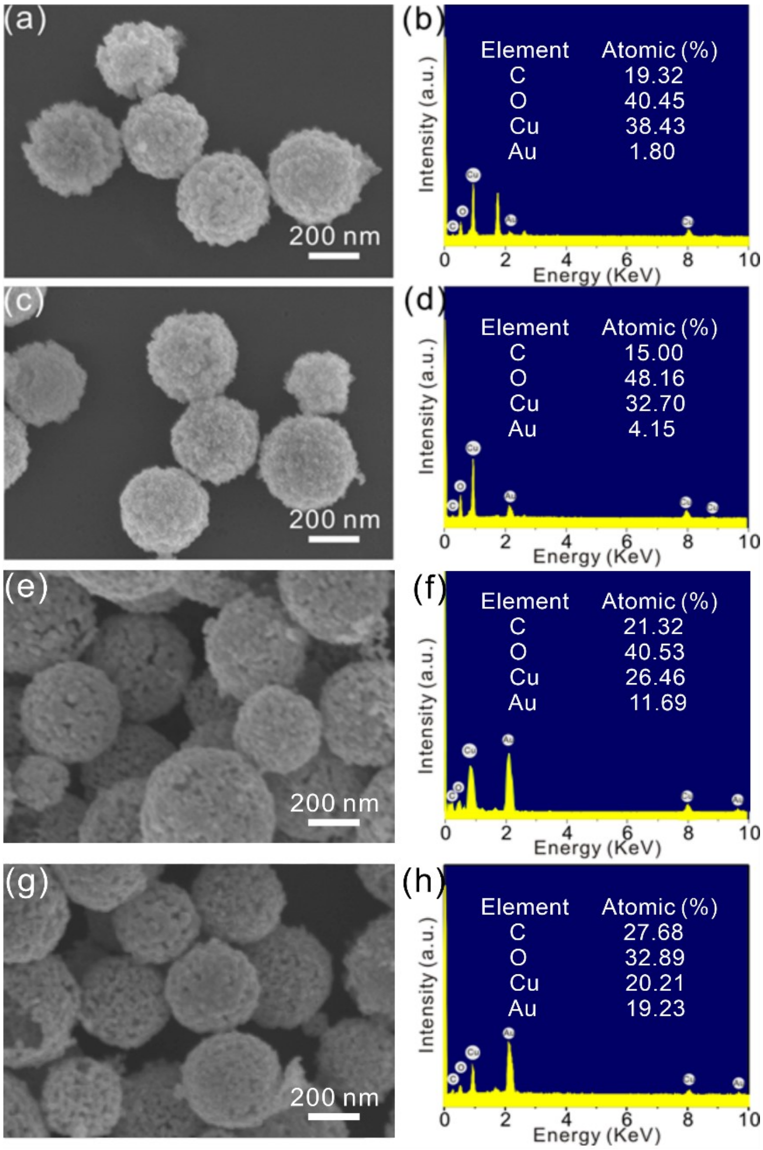

Fig. S1 (a, c, e and g) SEM images of Cu_xO/Au-2.5, Cu_xO/Au-5, Cu_xO/Au-10 and Cu_xO/Au-15, respectively. (b, d, f and h) EDS spectra of Cu_xO/Au-2.5, Cu_xO/Au-5, Cu_xO/Au-10 and Cu_xO/Au-15, respectively. The structural and compositional evolution of the Cu_xO/Au chimeric nanoparticles provides critical insights into the morphological changes and elemental distribution corresponding to varying concentrations of $HAuCl_4$ used during synthesis process. SEM images and EDS spectra for four representative samples, Cu_xO/Au-2.5, Cu_xO/Au-5, Cu_xO/Au-10, and Cu_xO/Au-15, offering a systematic understanding of how increased Au precursor concentration influences the composite formation. Beginning with Cu_xO/Au-2.5 (Fig. S1a), the SEM image reveals initial Au nanoparticle deposition on the Cu_xO surface. The corresponding EDS spectrum (Fig. S1b) confirms the presence of Cu, O, and Au, with a relatively low Au signal, consistent with

sparse decoration. As the $HAuCl_4$ concentration increases to 5 mM, which denoted as Cu_xO/Au-5, the SEM image (Fig. S1c) shows more extensive and dense Au coverage. The particulate deposits grow in number, suggesting progressive nucleation and coalescence of Au domains. The EDS data (Fig. S1d) reflect a noticeable increase in Au content, supporting the visual observation of enhanced Au deposition. With further increase in $HAuCl_4$ to 10 mM, which resulted in the formation of Cu_xO/Au-10, the composite undergoes significant transformation. The SEM image (Fig. S1e) displays extensive Au coverage, resulting in a core-shell-like morphology where the initial Cu_2O framework is largely obscured. Surface heterogeneity increases, and the particles exhibit aggregation and irregular contours. The EDS spectrum (Fig. S1f) shows a substantial rise in Au intensity relative to Cu, underscoring the dominance of Au in the composite's surface region. At the highest concentration of 15 mM，that is Cu_xO/Au-15, the structure evolves into a highly complex, porous, and cauliflower-like morphology (Fig. S1g). The original Cu_2O core is extensively consumed, leading in some cases to hollow or open frameworks due to the vigorous galvanic replacement process. This architectural degradation is corroborated by the EDS spectrum (Fig. S1h), which exhibits the strongest Au signal and a diminished Cu contribution, indicating substantial replacement of Cu by Au element. The EDS spectra collectively demonstrate a clear trend: as the $HAuCl_4$ concentration increases, the Au/Cu atomic ratio rises, confirming the controlled tuning of Au incorporation. This compositional shift is directly correlated with the morphological changes observed in the SEM images. These structural and compositional changes have profound implications for the material's functionality, particularly as a SERS substrate. After all, this figure offering a detailed view of the morphological and compositional evolution in the Cu_xO/Au series substrates, and which highlights the importance of optimizing synthesis conditions to achieve a balance between Au integration and structural preservation for maximum functional efficacy.

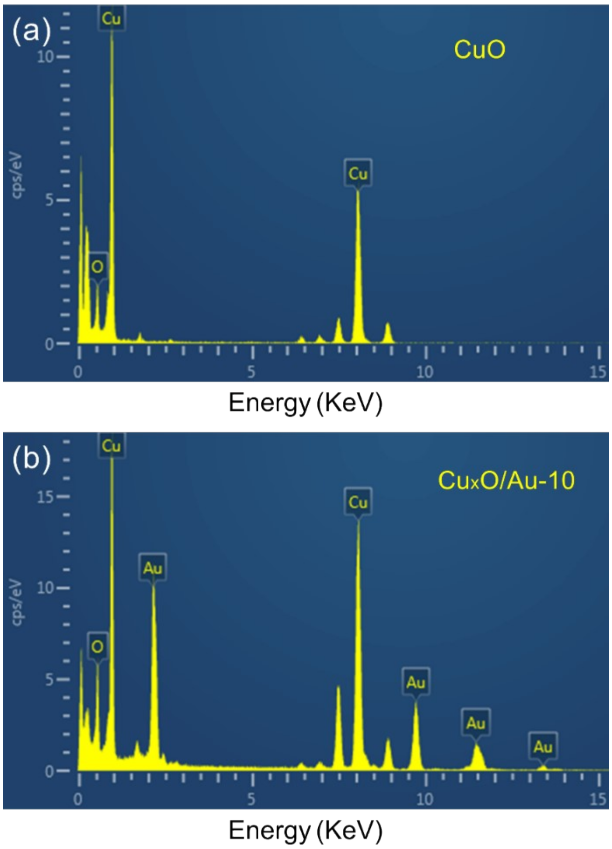

Fig. S2 Typical EDS spectra of (a) Cu_2O and (b) Cu_xO/Au-10, respectively.

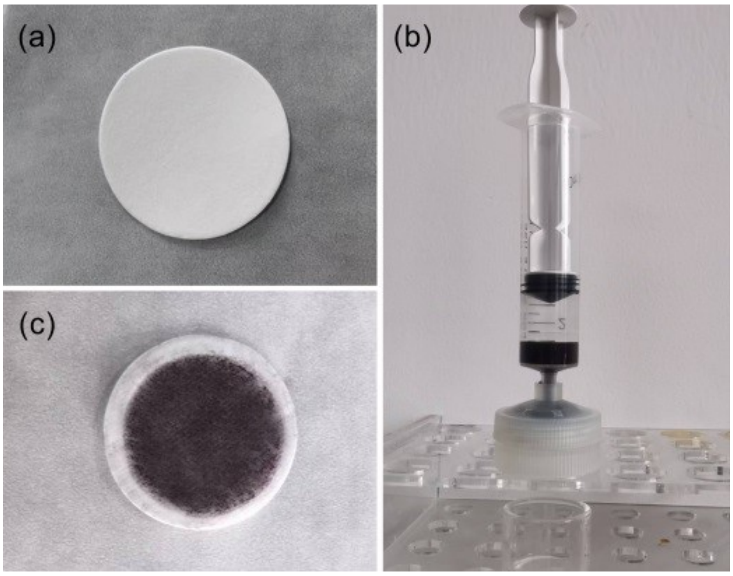

Fig. S3 (a) Blank polytetrafluoroethylene (PTFE) membrane. (b) Schematic diagram of the filter membrane loading process. (c) PTFE membrane loaded with Cu_xO/Au-10 chimeric micro-nanoparticles.

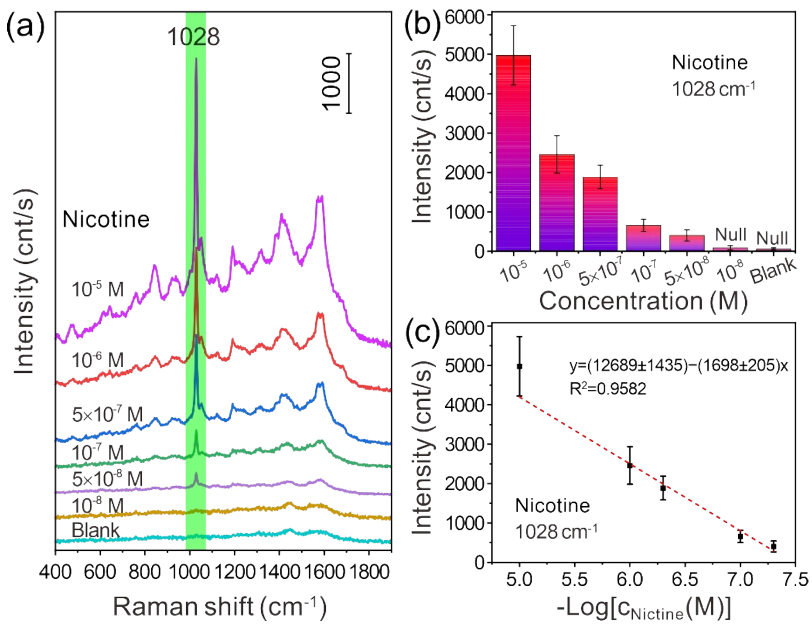

Fig.S4 (a) SERS spectra of nicotine at various concentrations based on Cu_xO/Au-10 substrates loaded in PTFE membrane. (b) Comparison of SERS signal intensities at 1028 cm^{-1} for different concentrations of nicotine solution. (c) The calibration plot for different concentrations of nicotine solution at 1028 cm^{-1}. Fig. S4a displays the SERS spectra obtained from Cu_xO/Au-10 substrates after incubation with nicotine solutions at varying concentrations. The spectra show characteristic Raman peaks of nicotine, with the intensity changes reflecting the concentration-dependent enhancement. Fig.S4b compares the SERS signal intensities at the 1028 cm^{-1}, which is the key vibrational mode associated with nicotine. This comparison highlights how the signal intensity varies with different nicotine concentrations, demonstrating that the substrate provides a reliable quantitative response. The results also reveal that the Cu_xO/Au-10 substrate achieved detectability of 5×10^{-8} M for nicotine. Fig. S4c illustrates the calibration plot derived from the data in (b), plotting signal intensity at 1028 cm^{-1} against nicotine concentration. The results demonstrate a clear positive correlation between the signal intensity and the concentration. In summary, Fig.S4 effectively validates the Cu_xO/Au-10 substrates as reliable SERS platforms for nicotine sensing. by correlating the results from Fig.S4 in the revised Supporting Information and Fig.5 in the revised manuscript, the approximate concentration range of nicotine in the aerosol can be estimated based on the SERS signal intensity. For traditional cigarettes, the nicotine concentration in the aerosol ranges from approximately 1×10^{-7} M to 5×10^{-7} M, while for HNB cigarettes, it ranges from about 6×10^{-8} M to 4×10^{-7} M.

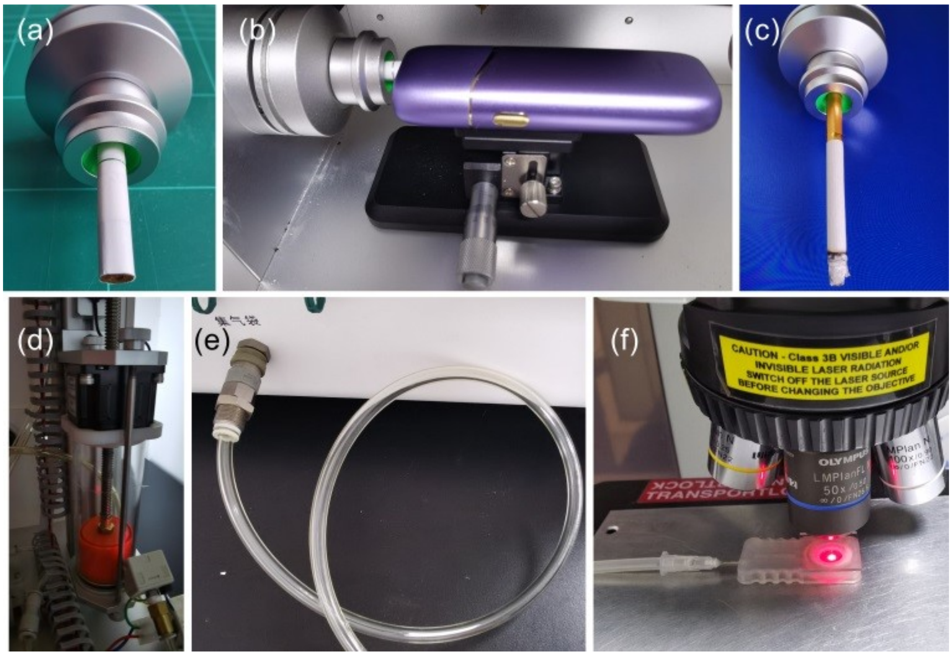

Fig.S5 Device design demonstration diagram: (a) HNB cigarette is placed in a simulated smoking mouthpiece (trap). (b) HNB cigarette is placed in a simulated smoking mouthpiece (trap) when heated by an electronic cigarette device. (c) Traditional cigarette is placed in a simulated smoking mouthpiece (trap) when lit. (d) Simulated smoking device (artificial suction pump). (e) Gas collection bag interface smoke outlet pipe. (f) PTFE membrane loaded with Cu_xO/Au-10 chimeric micro-nanoparticles is encapsulated in a microflow channel and placed on the Raman spectrometer workbench for SERS detection, with an excitation light wavelength of 633 nm.
